# Navigating veterans with an abnormal prostate cancer screening test: a quasi-experimental study

**DOI:** 10.1186/1472-6963-13-314

**Published:** 2013-08-15

**Authors:** Melissa A Simon, Narissa J Nonzee, June M McKoy, Dachao Liu, Thanh Ha Luu, Peter Byer, Elizabeth A Eklund, Elizabeth A Richey, Zhigang Wu, XinQi Dong, Alfred W Rademaker

**Affiliations:** 1 Robert H. Lurie Comprehensive Cancer Center of Northwestern University, Chicago, IL, USA; 2 Northwestern University Feinberg School of Medicine, Chicago, IL, USA; 3 Jesse Brown VA Medical Center, Chicago, IL, USA; 4 Rush University, Chicago, IL, USA; 5 Dartmouth College Geisel School of Medicine, Hanover, NH, USA; 6 University of Illinois at Chicago College of Medicine, Chicago, IL, USA

## Abstract

**Background:**

Prostate cancer disproportionately affects low-income and minority men. This study evaluates the impact of a patient navigation intervention on timeliness of diagnostic resolution and treatment initiation among veterans with an abnormal prostate cancer screen.

**Methods:**

Participants were enrolled between 2006 and 2010. The intervention involved a social worker and lay health worker navigation team that assisted patients in overcoming barriers to care. For navigated (n = 245) versus control (n = 245) participants, we evaluated rates of diagnostic resolution and treatment and adjusted for race, age, and Gleason score.

**Results:**

Of 490 participants, 68% were African American, 47% were ≥ 65 years old, and 35% had cancer. Among those with an abnormal screen, navigation did not have a significant effect on time to diagnostic resolution compared to controls (median days of 97 versus 111; adj. HR 1.17, 95% CI, 0.96-1.43, p = 0.12). On analysis of the period beyond 80 days, navigated men reached resolution faster than controls (median of 151 days versus 190 days; adj. HR 1.41, 95% CI, 1.07-1.86, p = 0.01). Among those with cancer, navigation did not have a significant effect on time to treatment initiation compared to controls (median of 93 days versus 87 days; adj. HR 1.15, 95% CI, 0.82-1.62, p = 0.41).

**Conclusion:**

Our navigation program did not significantly impact the overall time to resolution or treatment for men with prostate cancer compared to controls. The utility of navigation programs may extend beyond targeted navigation times, however, and future studies focusing on other outcomes measures are therefore needed.

## Background

Prostate cancer accounts for 28% of newly diagnosed cancers and is the second leading cause of cancer-related deaths among men in the United States [1]. Although prostate cancer screening remains controversial, five-and ten-year relative survival rates are highly favorable if prostate cancer is detected early [2]. Over the past 20 years, prostate cancer death rates have declined considerably, but progress has not been equally shared among all populations [1]. In particular, low-income men have increased risk of distant-stage prostate cancer [3], and African Americans have the highest prostate cancer mortality rate of any racial or ethnic group in the U.S. [4].

Multiple factors have been associated with delayed prostate cancer diagnosis, including health care system and physician distrust [5,6], lower household incomes [7], cancer misinformation [8], provider failure to facilitate timely follow-up [9], and culturally-influenced resistance to digital rectal examination (DRE) [10]. Patient navigation, a patient-centered intervention seeking to remove barriers to timely healthcare services [11], is one possible strategy for improving cancer outcomes–particularly among historically disadvantaged populations. In the 1990s, Dr. Harold Freeman established the first patient navigation program for low-income minority women in Harlem, New York. The findings suggested that navigation may increase rates and timeliness of diagnostic resolution following an abnormal breast cancer screening finding [12]. Based on the promising benefits of this model, the National Cancer Institute’s (NCI) Center to Reduce Cancer Health Disparities (CRCHD) and American Cancer Society (ACS) funded nine sites for the Patient Navigation Research Program (PNRP) [13], the first multi-center study to critically examine and demonstrate the efficacy of navigation [14-19].

Most navigation programs have emerged in outpatient clinical settings and have focused on breast, cervical, or colorectal cancer; few have reported on prostate cancer outcomes [11,20-25]. Only one nurse navigation program has evaluated time to treatment initiation at a Veterans Affairs (VA) hospital, but used historical controls and found it challenging to isolate the effects of navigation from other systems changes simultaneously implemented within a larger VA process improvement project [26]. One combined prostate cancer education and navigator program demonstrated increased prostate cancer screening rates among African Americans [27], and a PNRP site reported navigation’s effect on diagnostic resolution of a prostate screening abnormality [19]. In the Chicago PNRP, we uniquely implemented a navigation intervention among predominantly low-income and minority men at a VA, where access to care barriers are diminished. We evaluated time to diagnostic resolution and treatment initiation among navigated versus control patients with an abnormal prostate cancer screen.

## Methods

### Setting

The study took place at a Chicago VA, an equal access system in an urban medical district. The tertiary care facility and its four community-based outpatient clinics provide care to approximately 58,000 veterans in Chicago, the county (Cook) in which Chicago resides, and six counties in northwestern Indiana. A large proportion of veterans in Illinois are African American, and nearly half in Cook County are over age 65 [28].

### Study design

Our protocol has been described in detail previously [29]. Briefly, the study used a quasi-experimental design. Eligible patients were identified through electronic medical records. Patients were selected to receive navigation if they met inclusion criteria and presented to the urology clinic on a clinic day designated for the navigation intervention (n = 245). All patients who presented on a clinic day designated for non-navigation and met inclusion criteria were identified as controls. Control subjects received usual care and comprised a consecutive and concurrent records-based sample with a one-to-one match goal (n = 245). The clinic was staffed with rotating resident physicians and stable supervising attending physicians and nurses who remained constant across navigated and non-navigated clinic days.

### Eligibility and recruitment

Our study enrolled patients from November 2006 through April 2010. All patients who presented to the VA urology clinic for follow-up of an abnormal prostate cancer screening test (prostate specific antigen (PSA) ≥ 4.0 ng/mL, PSA < 4.0 ng/mL with an abnormal PSA velocity defined by a physician, or abnormal DRE) and who were recommended for a biopsy were eligible. We excluded patients if they were less than age 18, institutionalized, cognitively impaired, previously navigated for an abnormal cancer finding, or treated for any cancer except non-melanoma cancer within the past five years. Our study team approached patients for participation following provider referral on the first follow-up visit in the urology clinic. We obtained written informed consent from navigated patients, and the Institutional Review Board (IRB) granted a waiver of authorization for our control sample. The Northwestern University IRB, Jesse Brown VA Medical Center, and the Collaborative IRB at the University of Illinois at Chicago approved the study.

### Patient navigation

The patient navigation team comprised two full-time male navigators: one social worker and one lay health worker [29]. Navigators participated in local training, monthly state-level ACS trainings, and national annual PNRP training conferences relative to navigator roles, health disparities, cancer knowledge, cultural diversity, and communication [30].

Upon enrollment, navigators conducted face-to-face interviews with patients to identify potential barriers to care, and follow-up assistance occurred in person or over the phone until diagnostic resolution for those without prostate cancer, primary therapy completion for those with cancer, or until the end of the study. The number of contacts varied according to need, but at minimum, each patient received appointment reminder calls one month and ten days prior to his scheduled biopsy appointment to ensure compliance with preparatory instructions [29]. Additional navigation activities included social support, facilitation of patient-provider communication, transportation coordination, and patient education. Navigators also often accompanied patients with prostate cancer to treatment appointments and connected each with a cancer survivor through an established ACS program.

### Data collection

We collected self-reported demographic data at baseline in the navigated arm and obtained them through chart audit in the control arm. Navigators documented each encounter, barrier, and action taken on standardized tracking logs developed based on the needs assessment of our targeted population. Our needs assessment involved interviews with key hospital staff and patients who delayed abnormal screen follow-up and focused on identification of personal and system barriers to care [29]. Using the VA's comprehensive computerized patient record system, trained research assistants performed chart audits for clinical outcomes data following national PNRP guidelines [13]. Teams of researchers (N.N., P.B., T.L., and E.R.) independently abstracted data on a subset of the entire sample and iteratively compared results until they achieved consistent agreement; clinical co-investigators (M.S. and J.M.) adjudicated discrepancies. The project and data managers thoroughly reviewed data completeness and cleaned entry errors on a quarterly basis.

### Outcome measures

The primary outcome for the prostate screening group was time from abnormal screening test to diagnostic resolution (Time 1). For the prostate cancer diagnosis group, the primary outcome was time from diagnostic resolution to treatment initiation (Time 2). These events had to occur before 365 days from abnormal screen for Time 1 or 365 days from diagnosis for Time 2 to be considered events in the statistical analysis. Otherwise, they were censored at 365 days. Abnormal screening was defined as the date of the abnormal prostate screening test (PSA or DRE) prompting referral for follow-up, and definitive diagnosis was defined as the date of the diagnostic test (transrectal ultrasound (TRUS)-guided biopsy or follow-up PSA test) resolving the screening test into a cancer diagnosis or non-cancer diagnosis (negative biopsy on pathology or normal PSA level/velocity for those who resolved with a follow-up PSA test). Treatment initiation was defined as the first date of treatment receipt with single or combination therapy (surgery, radiation, hormonal therapy, cryotherapy, or decision to watch and wait).

### Statistical analysis

Based on preliminary data and our group’s clinical experience with veterans with prostate cancer, we estimated that the mean time from abnormal screen to diagnostic resolution (Time 1) in the control group would be 103 days, and the mean time from cancer diagnosis to treatment initiation (Time 2) in cancer patients would be 128 days. The initial power calculation indicated that for Time 1, 300 patients per group would have 80% power to detect a mean Time 1 of 86 days in the navigated group, and that 120 cancer patients per group would have 80% power to detect a mean Time 2 of 90 days in the navigated group. Since only 245 instead of 300 patients were enrolled per group for Time 1, the actual effect size for Time 1 that was detectable with 80% power was 103 days versus 84 days. Since only 85 instead of 120 patients were enrolled per group for Time 2, the actual effect size for Time 2 that was detectable with 80% power was 128 days versus 82 days. Two-tailed tests and a Type I error rate of 5% were assumed.

All statistical analyses were conducted using SAS version 9.2 (SAS Institute, Inc., Cary, North Carolina) [31]. For categorical variables, frequencies and percentages were calculated by group and for the whole sample. Comparisons between groups were made using Fisher’s exact test. Unadjusted and adjusted time-to-event analyses were done for Time 1 and Time 2. For unadjusted analyses, Kaplan-Meier curves were calculated, median times (25th percentile for one analysis since the median was not attained) within groups were determined from these curves, and curves were compared between groups using the log-rank test. For adjusted analyses, proportional hazards regression was used. The proportional hazards assumption was assessed by inclusion of a group by log-time interaction term. For Time 1, age (< 65, ≥ 65), and race (African American, non-African American) were used as covariates. Baseline absolute PSA value was not included in the model, as nearly one-fifth of patients were eligible based on an abnormal DRE or PSA velocity. For Time 2, age (< 65, ≥ 65), race (African American, non-African American), and total Gleason score (≤ 6, > 6) were used as covariates. Adjusted hazard ratios (HR) and 95% confidence intervals are reported, together with a p-value testing whether the HR was 1.0. A HR greater than 1 indicated that the outcome occurred earlier in the navigated group compared with the control group. A HR less than 1 indicated that the outcome occurred later in the navigated group compared with the control group. Kaplan-Meier curves showed a crossover time point, an indication that the proportional hazards assumption might be violated; thus, time to event was analyzed separately before the cross-over period in each analysis. A significant group by log-time interaction (p = 0.0009) indicated the proportional hazards assumption did not hold for Time 1. For Time 1, navigated and control groups were then compared (a) using all participants in the analysis and censoring everyone with a follow-up time greater than 80 days and (b) using only those participants with follow-up greater than 80 days. The proportional hazards assumption did hold for Time 2. For Time 2, navigated and control groups were compared within subgroups determined by: (a) Gleason score (≤ 6 vs. > 6); (b) treatment type; and (c) receipt of consultation beyond a urologist, given that multiple specialist visits could delay treatment initiation.

## Results

### Study participants

**Figure 1:**
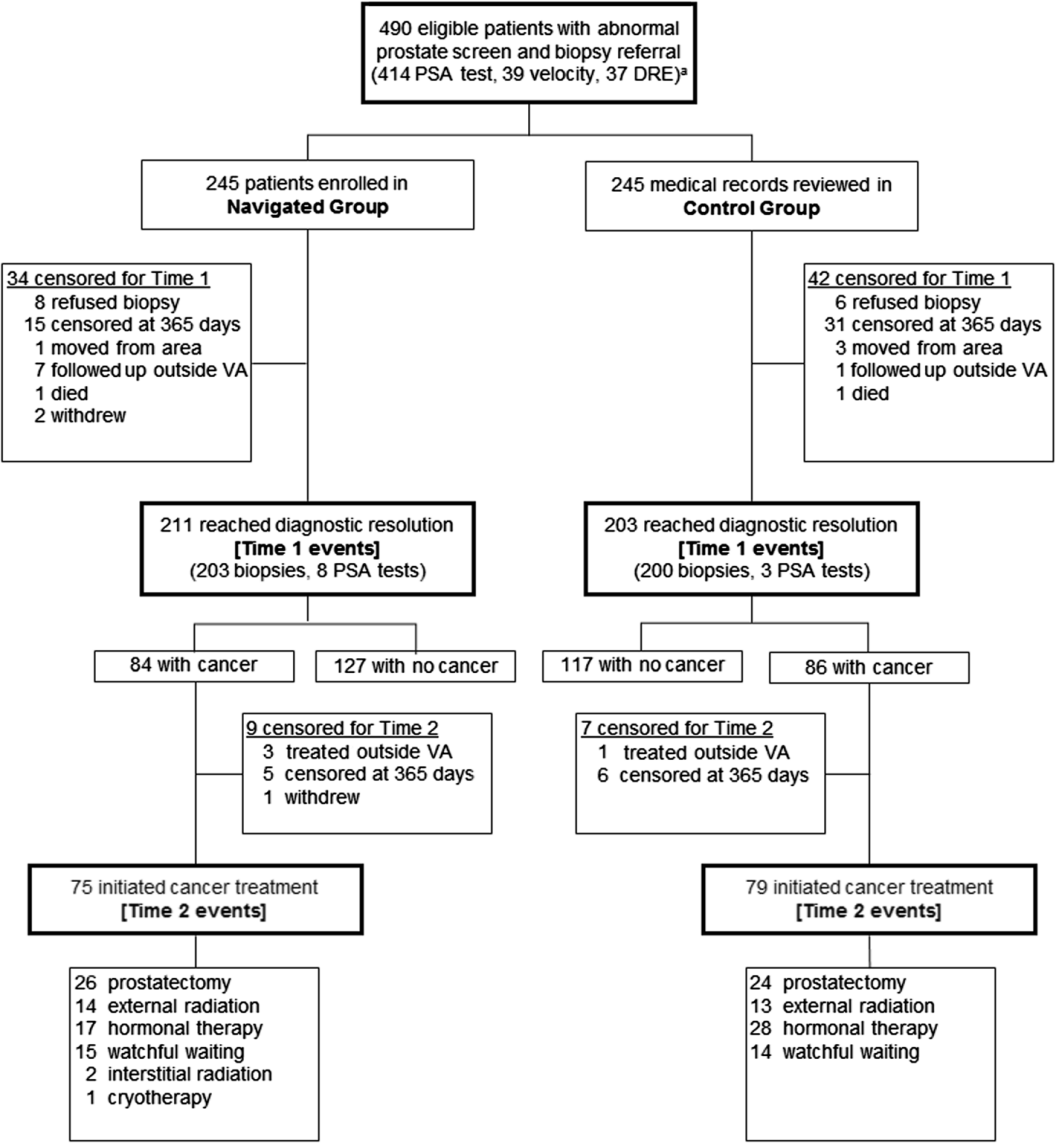
**Prostate navigation study flow chart.** The chart shows the flow of patients through the study, including the proportion who reached a diagnostic resolution and treatment initiation and those who were censored. Abbreviations: PSA, prostate specific antigen; DRE, digital rectal exam; VA, Veterans Affairs hospital; ^a^Eligibility types: (1) elevated PSA, PSA ≥ 4.0 nanograms/milliliter (ng/mL); (2) PSA < 4.0 ng/mL with an abnormal velocity; (3) abnormal DRE.

Figure 1 presents a flow chart of patient progression through the study. Of the 490 study participants, 245 were enrolled in each of the control and patient navigation intervention groups. For both Time 1 and Time 2, reasons for censoring are tabulated for each study group. Of the 490 patients, 414 reached diagnostic resolution and are considered events in the time-to-event analysis of Time 1. Of the 170 patients diagnosed with cancer, 154 initiated treatment and are considered events in the analysis of Time 2.

Baseline participant characteristics are reported in Table 1. Almost all patients (92%) were eligible based on an abnormal PSA test, 47% were at least 65 years old, two-thirds were African American, and 86% had a low Charlson Comorbidity Index [32] score (0-2). There were significant differences between control and navigated participants in age (p < 0.05), race/ethnicity (p < 0.0001), and baseline PSA (p = 0.008). Among the 170 patients diagnosed with cancer, half were at least 65 years old, 69% were African American, and 57% had a Gleason score greater than 3 + 3 (Table 2). A total of 213 patients refused to participate in navigation, many citing lack of time as the reason.

**Table 1:** Patient characteristics, all subjects (N = 490)

	**Navigated (N = 245)**	**Control (N = 245)**	**p-value**
	***N *(%)**	***N *(%)**	
**Age**			0.046
< 65 years old	142 (58)	119 (49)	
≥ 65 years old	103 (42)	126 (51)	
Range	48-79	42-92	
**Race/ethnicity**			<0.0001
African American	155 (63)	177 (72)	
White	68 (28)	37 (15)	
Other	20 (8)	7 (3)	
Not reported	2 (1)	24 (10)	
**Comorbidities (CCI Score)**			0.72
0	96 (39)	90 (37)	
1-2	114 (47)	123 (50)	
> 2	35 (14)	32 (13)	
**Screening eligibility**			0.058
PSA test (≥ 4.0 ng/mL)	204 (83)	210 (86)	
Abnormal PSA velocity	26 (11)	13 (5)	
Abnormal (DRE)	15 (6)	22 (9)	
**Baseline PSA**			0.011
< 4 ng/mL^a^	26 (11)	11 (5)	
4 ng/mL - 10 ng/mL	164 (71)	154 (69)	
> 10 ng/mL	40 (17)	58 (26)	
Missing (Eligible by DRE)	15 (6)	22 (9)	
**Cancer diagnosis**			0.54
No cancer/resolved with PSA test	127 (52)	117 (48)	
Cancer	84 (34)	86 (35)	
Unresolved	34 (14)	42 (17)	

*Abbreviations:* CCI, Charlson Comorbidity Index; PSA, prostate specific antigen; ng/mL, nanogram per milliliter; DRE, digital rectal exam.

^a^Values may not sum to all subjects who enrolled on abnormal PSA velocity due to rounded PSA value.

**Table 2:** Patient characteristics, subjects with cancer (N = 170)

	**Navigated (N = 84)**	**Control (N = 86)**	**p-value**
	***N *(%)**	***N *(%)**	
**Age**			0.17
< 65 years old	47 (56)	39 (45)	
≥ 65 years old	37 (44)	47 (55)	
Range	50-79	43-92	
**Race/ethnicity**			0.011
African American	53 (63)	64 (74)	
White	23 (27)	13 (15)	
Other	7 (8)	2 (2)	
Not reported	1 (1)	7 (8)	
**Gleason score**			0.008
≤ 3 + 3	45 (54)	28 (33)	
> 3 + 3	39 (46)	58 (67)	
**Consultation beyond Urology**^a^			0.36
Yes	35 (42)	42 (49)	
No	49 (58)	44 (51)	
**Primary Therapy**			0.45
Prostatectomy	26 (30)	24 (28)	
External Radiation Therapy	14 (17)	13 (15)	
Hormone Therapy	17 (20)	28 (33)	
Watchful Waiting	15 (18)	14 (28)	
Interstitial Radiation Therapy	2 (2)	0 (0)	
Cryotherapy	1 (1)	0 (0)	
Treatment not initiated	9 (11)	7 (8)	

^a^Consultation with radiation oncologist, medical oncologist, or surgeon.

### Time to diagnostic resolution

Among our total sample, 85% reached a diagnostic resolution (50% with a negative resolution based on a negative biopsy or repeat PSA test; 35% with a positive diagnosis) (Table 1). Seventy-six (16%) were unresolved (Figure 1). Resolution rates were comparable between the study groups.

**Figure 2:**
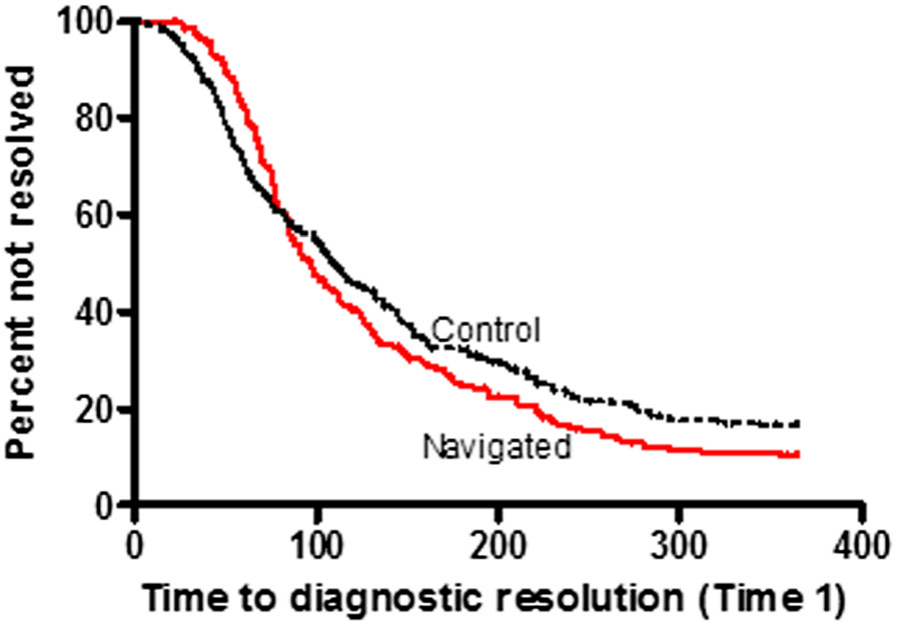
**Time to diagnostic resolution (Time 1) Kaplan-Meier survival curves.** The curves illustrate the proportion of navigated and control patients who did not resolve an abnormal prostate cancer screening test within one year. Time (days) to event (resolution) was analyzed separately before and after the crossover time point.

With respect to Time 1, navigated patients had a diagnostic test completion rate of 211 events among 245 patients versus 203 events among 245 control patients. Navigation did not significantly affect time to diagnostic resolution compared to controls (median days of 97 versus 111; adj. HR 1.17, 95% CI, 0.96 – 1.43, p = 0.12). Kaplan-Meier (Figure 2) curves show a crossover time point; thus, time to event was analyzed separately before 80 days (using the total sample size and censoring follow-up times greater than 80 days) and after the 80 days (using those with follow-up greater than 80 days). Prior to 80 days, the 25th percentile of the time to diagnostic resolution was not significantly different between the navigated and control groups (25th percentile of 68 days versus 55 days, p = 0.45, Table 3). The adjusted HR was 0.91 (p = 0.53). Beyond 80 days, median time to diagnostic resolution was significantly shorter among the navigated group compared to the control group (median of 151 days versus 190 days, p = 0.015). The adjusted HR was 1.41 (p = 0.01).

**Table 3:** Summary of analyses of time to diagnostic resolution (Time 1) and time to treatment initiation (Time 2)

**Analysis**	**Category**	**Study arm**	**Sample size**	**Event**	**Median days**	**Log rank p-value**	**Adj. H.R.**^**a**^	**Adj. 95% C.I.**	**Adj. p-value**
**Time 1: Time from abnormal screening to diagnostic resolution**									
Time 1 stratified by diagnostic resolution time interval	0-80 days	Navigated	245	95 (38.8%)	68^b^		0.91		0.53
		Control	245	96 (39.2%)	55^b^	0.45		0.67 – 1.23	
	81-365 days	Navigated	146	116 (79.4%)	151		1.41		0.01
		Control	148	107 (72.3%)	190	0.015		1.07 - 1.86	
**Time 2: Time from diagnostic resolution to treatment initiation**									
Time 2 overall	0-365 days	Navigated	84	75 (89.3%)	93		1.15		0.41
		Control	86	79 (91.9%)	87	0.36		0.82 - 1.62	

Abbreviations: *H.R.* hazard ratio.

^a^For Time 1, age (< 65, ≥ 65) and race (African American, non-African American) were used as covariates. For Time 2, age (< 65, ≥ 65), race (African American, non-African American), and Gleason score (≤ 6, > 6) were used as covariates.

^b^25th percentile since median not attained.

In the analysis of Time 1 by race (African American, White), outcomes were similar to overall results. Prior to 80 days, the 25th percentile of the time to diagnostic resolution was similar in the navigated and control groups within each racial group (data not shown). Beyond 80 days, the median time to diagnostic resolution was shorter among the navigated group within each racial group, and significant among White participants (median of 122 days versus 179 days, p = 0.047).

### Time to treatment initiation

**Figure 3:**
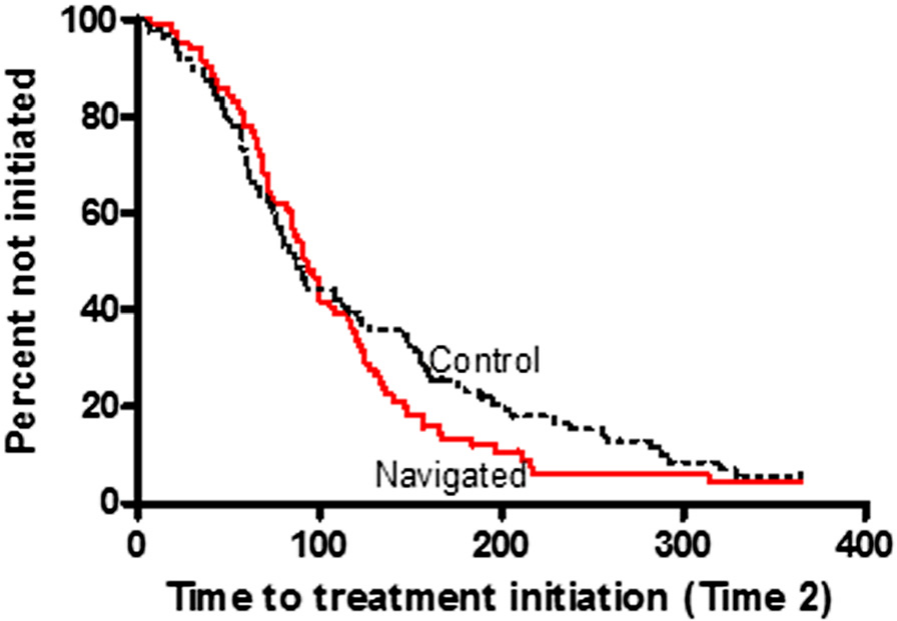
**Time to Treatment Initiation (Time 2) Kaplan-Meier Survival Curves.** The curves illustrate the proportion of navigated and control patients who did not initiate prostate cancer treatment within one year of diagnosis. Time (days) to event (treatment initiation) was analyzed separately before and after the crossover time point.

Among patients diagnosed with cancer, 16 (9%) did not initiate treatment after diagnosis (Table 2 and Figure 1). With respect to Time 2, navigated patients had a treatment initiation rate of 75 events among 84 patients versus 79 events among 86 control patients (Table 3). Median time to treatment initiation was 93 days in the navigated group and 87 days in the control group, p = 0.36. The adjusted HR was 1.15 (p = 0.41). Kaplan-Meier curves are illustrated in Figure 3.

In the analysis of Time 2 by race (African American, White), results were similar to the overall results: no navigated-control differences in time to treatment initiation were observed within either race group. In addition, we found no difference in navigated versus control group median days to treatment initiation with respect to Gleason score or consultation beyond a urologist (data not shown). With respect to treatment types, no difference was observed except among men whose primary therapy was radiation, where time to treatment initiation was significantly lower for navigated versus control group participants (median of 95 days versus 120 days, p = 0.05).

## Discussion

In our study, we found no overall effect of patient navigation on time to diagnostic resolution or time to treatment initiation among veterans with an abnormal prostate cancer screening test or cancer diagnosis, respectively, regardless of race.

Several possible explanations for our findings exist. First, our intervention may have missed the crucial time point to begin navigation. In a randomized navigation trial based at public hospitals, Bastani et al. found that women who missed their first appointment following an abnormal breast screening had no chance of timely diagnostic resolution [33]. She posited that clinic staff may have been more invested in ensuring that women who kept their first appointment understood the follow-up process, resulting in better appointment adherence downstream. Men in our study had already kept their first follow-up appointment in the urology clinic, the first opportunity presented to obtain written informed consent, which may have similarly accounted for the absence of overall navigation effect. Our VA also included other well-established support mechanisms, such as the urology clinic nurse practitioner who provided prostate cancer education and volunteer clinic staff who performed appointment reminders. Finally, though we anticipated gender-matched navigation would facilitate communication, male patients have demonstrated less receptivity to social support in the clinical setting than women [34], which additionally could have diminished the intervention’s effect.

Though the overall finding for time to resolution was negative, navigated patients who did not reach diagnostic resolution within the first 80-day period resolved their screening test faster than controls. This shortened time may be attributed to factors inherent to navigation: the establishment of trust, access to education and information, and decreased anxiety. Men who delay diagnostic resolution beyond a 3-month period may merit outreach via patient navigation. In subgroup analyses among participants with prostate cancer, navigated men whose primary therapy was radiation had a shorter time to treatment than controls. Further research to better delineate the navigators’ role in supporting men through the complex treatment decision making process is needed.

Study limitations include, first, a lack of individual randomization to participate in navigation. We initially planned to implement the program at two similar VA outpatient centers, one designated as the navigation site and the other as the control site; however, a hospital merger just prior to accrual made this design logistically unfeasible. We ultimately chose to assign navigation based on clinic day to allow for minimal disruption to patient care during clinic reconfiguration and to reduce contamination between study arms at the single clinic site. While enrolling navigated patients and contemporaneous controls on designated clinic days could lead to selection bias, all patients were selected from the same clinic at one VA location and cared for by physicians with similar practice patterns. Second, we acknowledge that the differences in race between our control and navigated groups may have been due to use of administrative data versus self-reported race/ethnicity data, respectively, in each arm—two different methods showing low agreement in other VA studies [35,36]. Third, our findings may not be generalizabile to non-VA hospitals and non-VA male populations. Fourth, our outcome measures focused on time may not have targeted the true value of prostate cancer navigation. Finally, referral bias remains a concern, since patients referred to a study tend to be more motivated about their health.

Despite limitations, this study is the first controlled navigation intervention that elucidates prostate cancer outcomes at a VA. The Denver PNRP implemented their intervention in an integrated safety-net health care system and found that prostate cancer navigation shortened time to diagnostic resolution, with borderline significant results attributed to a small sample size and potential reduced screening practice among older age groups [19]. By contrast, our VA routinely screened veterans including older men and those with chronically elevated PSA tests, which may have conferred less urgency to fast-track them through the referral process. Furthermore, insufficient evidence supporting negative long-term effects of diagnostic or treatment delays on biochemical progression [37,38] or survival [39] could have also reduced urgency within our cancer group.

## Conclusions

In summary, our study indicated that patient navigation did not significantly reduce time to diagnostic resolution among men with an abnormal prostate cancer screen or treatment initiation among men with prostate cancer. Targeting patients who miss their first follow-up appointment after identification of abnormal finding may have more optimally impacted time-related outcomes. Notably, prostate cancer is the second most frequently diagnosed cancer among American men yet holds the most controversial track record for screening and has a range of treatment options, including the choice to not pursue treatment. It is therefore imperative that emerging navigation programs consider their impact on additional parameters beyond timelier care, such as quality of life and treatment decision making, to address prostate cancer disparities.

## Abbreviations

DRE: Digital rectal exam; NCI: National Cancer Institute; ACS: American Cancer Society; PNRP: Patient Navigation Research Program; VA: Veterans Affairs; PSA: Prostate specific antigen; IRB: Institutional Review Board; TRUS: Transrectal ultrasound; HR: Hazard ratio; HR: Hazard ratio; CCI: Charlson Comorbidity Index.

## Competing interests

The authors declare that they have no competing interests.

## Authors’ contributions

MS, NN, JM, and AR contributed to the study design and interpretation of data. MS, NN, and AR drafted the manuscript, and JM and EE revised it for important intellectual content. NN coordinated the study, and AR and DL performed the statistical analyses. PB, NN, TL, ER, and ZW contributed to participant recruitment and data collection and management. All authors participated in manuscript revision and read and approved the final manuscript.

## Pre-publication history

The pre-publication history for this paper can be accessed here:

http://www.biomedcentral.com/1472-6963/13/314/prepub
